# Epidermal growth factor alleviates the negative impact of urea on frozen-thawed bovine sperm, but the subsequent developmental competence is compromised

**DOI:** 10.1038/s41598-021-83929-z

**Published:** 2021-02-25

**Authors:** Rasoul Kowsar, Shahrzad Ronasi, Nima Sadeghi, Khaled Sadeghi, Akio Miyamoto

**Affiliations:** 1 Department of Animal Sciences, College of Agriculture, Isfahan University of Technology, 84156-83111 Isfahan, Iran; 2 Global Agromedicine Research Center (GAMRC), Obihiro University of Agriculture and Veterinary Medicine, Obihiro, Hokkaido 080-8555 Japan; 3 FKA, Animal Husbandry and Agriculture Co, Isfahan, Iran

**Keywords:** Predictive markers, Embryology

## Abstract

Upon insemination, sperm cells are exposed to components of the female reproductive tract (FRT) fluids, such as urea and epidermal growth factor (EGF). It has been shown that both urea and EGF use EGF receptor signaling and produce reactive oxygen species (ROS) that are required at certain levels for sperm capacitation and acrosome reaction. We therefore hypothesized that during bovine sperm capacitation, a high level of urea and EGF could interfere with sperm function through overproduction of ROS. High-level urea (40 mg/dl urea is equal to 18.8 mg/dl of blood urea nitrogen) significantly increased ROS production and TUNEL-positive sperm (sperm DNA fragmentation, sDF) percentage, but decreased HOS test score, progressive motility, acrosome reaction and capacitation. The EGF reversed the negative effects of urea on all sperm parameters, with the exception of ROS production and DNA fragmentation, which were higher in urea-EGF-incubated sperm than in control-sperm. The developmental competence of oocytes inseminated with urea-EGF-incubated sperm was significantly reduced compared to the control. A close association of ROS production or sDF with 0-pronuclear and sperm non-capacitation rates was found in the network analysis. In conclusion, EGF enhanced urea-reduced sperm motility; however, it failed to reduce urea-increased sperm ROS or sDF levels and to enhance subsequent oocyte competence. The data suggests that any study to improve sperm quality should be followed by a follow-up assessment of the fertilization outcome.

## Introduction

The use of artificial insemination (AI) techniques, along with the use of progeny-tested bulls, has been attributed to the development of animal reproduction, increased milk productions and, as a result, increased income for the dairy business^1^. However, the rate of pregnancy in dairy cows is not as effective as in beef cattle using fixed-time AI programs due to lactation-related reproductive deficiency, postpartum anestrus, uterine health and embryo loss^2^. Postpartum reproductive tract of lactating dairy cows has been shown to be less likely to support reproduction. This can lead to lower rates of pregnancy in dairy cows^3^. In addition, frozen-thawed sperm may have a condition in the female reproductive tract (FRT), i.e., elevated levels of urea and epidermal growth factor (EGF), which may decrease sperm fertility to a higher degree. Therefore, we tried to investigate some of the decreases in sperm fertility caused by factors that may be present in the FRT environment in this study.

In order to be able to fertilize an oocyte, sperm cells need to reside in the FRT for a few hours and undergo biochemical changes, such as capacitation and acrosome reaction^4^. Various factors, such as EGF, are normally present in FRT fluids that contribute to sperm acrosome reaction^5–7^*.* Ford^8^ reported that reactive oxygen species (ROS) promote sperm capacitation at physiological concentrations by increasing cAMP synthesis and inhibiting tyrosine phosphatase in bulls and human sperm. Bovine sperm cells express the EGF receptor (EGFR) which has been shown to contribute to sperm physiology, e.g. ROS production, capacitation, and acrosome reaction^6–9^.

The levels of urea in the blood and reproductive fluids of healthy dairy cows are closely linked to each other (R = 0.97) and the decreased fertility rate^10,11^. High-protein diets, 17–19% crude protein^12^, are fed to sustain the milk production potential of high-producing dairy cows. This can, in turn, lead to elevated blood urea nitrogen (BUN) levels and increased urea levels in FRT fluids, such as oviductal fluids^13^. Several studies have investigated the mechanisms by which urea can affect fertility in dairy cows, but little evidence exists on the developmental competence of oocytes inseminated with urea-exposed sperm cells^14^. For example, we reported that high levels of urea disrupted the oviduct epithelial cell monolayer and reduced the viability of bovine cumulus cells^15–17^. We also documented that urea-incubated oocytes had a lower rate of developmental competence and higher more amino acid depletion during in vitro maturation (IVM)^17,18^. It has been shown that urea uses EGFR transactivation and induces the expression of immediate-early genes^19^ and, as a toxic molecule, can induce ROS production in a variety of cells, such as oocytes^20^. There is no evidence that urea influences sperm production of ROS, but urea has been shown to cause toxicity by several mechanisms, including oxidative stress^21^. In this study, therefore, we expected that urea would cause an overproduction of ROS, which could lead to a reduction in sperm motility and the integrity of sperm DNA^9^.

We, therefore, hypothesized that frozen-thawed sperm cells would produce more ROS in the presence of urea and EGF, which could alter the quality of sperm and decrease subsequent developmental competence. In addition, using network analysis, we sought to establish a connection between ROS production, sperm DNA fragmentation (sDF), sperm quality and fertilization rate. In addition, the predictive power and cut-off point of ROS production/sDF were tested for high-quality sperm using the area under the receiver operating characteristic (ROC) curve (AUC).

## Results

### Determination of the effective doses of EGF and urea

**Figure 1:**
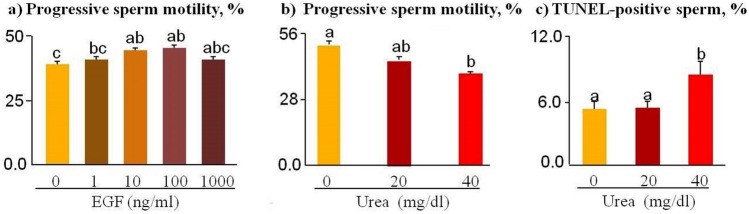
Determination of the effective dose of EGF and urea. Sperm cells were incubated for 30 min with different doses of EGF (0, 1, 10, 100, and 1000 ng/ml) and urea (0, 20, and 40 mg/dl), followed by progressive sperm motility (CASA) and DNA fragmentation (TUNEL test, n = 5) studies. The data was analyzed using the Tukey test. Numerical values are shown as mean ± SEM. Different letters (a, b, c) indicate significant differences between treatments at *P* < 0.05.

Frozen-thawed sperm cells were incubated for 30 min with different doses of EGF (0, 1, 10, 100, and 1000 ng/ml) and then progressive sperm motility was measured to assess the effective dose of EGF (Fig. 1a). It was noted that 10 ng/ml of EGF increased progressive sperm motility by (p < 0.05). 10 ng/ml of EGF was then selected for the next experiments.

In order to determine the effective dose of urea, sperm cells were incubated for 30 min with different levels of urea (0, 20, and 40 mg/dl), and progressive motility (Fig. 1b) and sDF were evaluated (percentage of TUNEL positive sperm, Fig. 1c). Results showed that, 40 mg/dl of urea increased sDF and decreased progressive sperm motility, respectively (p < 0.05). Therefore, 40 mg/dl of urea was selected for the next experiment**.**

### EGF abrogates urea-reduced quality of bovine sperm

**Figure 2:**
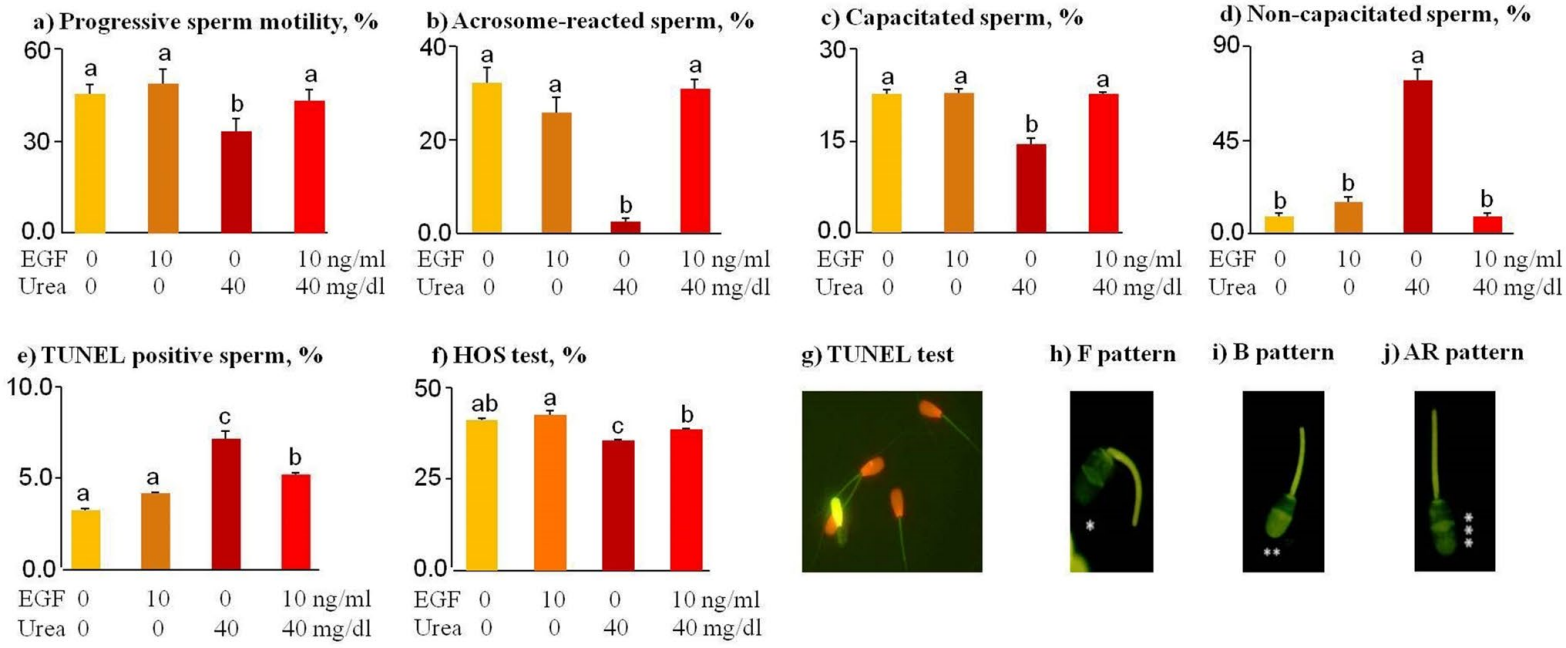
Bovine sperm cells were pre-incubated for 30 min with various levels of urea (0, 20, and 40 mg/dl) in the presence or absence of EGF (10 ng/ml). (**a**) Progressive sperm motility (n = 5) was determined using the CASA method. (**b**–**d**) Different patterns of chlortetracycline (CTC) staining in bovine sperm during capacitation process (n = 5). (**e**) DNA fragmentation (%) was determined using the TUNEL test (n = 5). (**f**) hypo-osmotic swelling (HOS) scores in bovine sperm (n = 4). (**g**) The sperm cells without DNA fragmentation were in red color and the sperm cells with DNA fragmentation (TUNEL-positive cells) were visible in green color. The TUNEL test was performed five times (n = 5) and at least 400 sperm cells were counted in each of the 10 fields of vision at each time; (**h**) *Represents F pattern (corresponds to non-capacitated sperm); (**i**) **Represents B pattern (corresponds to capacitated sperm); (**j**) ***Represents AR pattern (corresponds to acrosome-reacted sperm). The data was analyzed using the Tukey test. Numerical values are shown as mean ± SEM. Different letters (a, b, c) indicate significant differences between treatments at *P* < 0.05.

Compared to the control and EGF groups, high-level urea (40 mg/dl) significantly decreased the rate of progressive sperm motility (*p* < 0.05), acrosome reaction (*p* = 0.03) and sperm capacitation (*p* < 0.001, Fig. 2a–c). High-level urea increased (*p* < 0.01) the percentage of non-capacitated sperm (F pattern, Fig. 2d) and the percentage of TUNEL-positive sperm (sDF, Fig. 2e) compared to the control and EGF groups. Unexpectedly, experimental treatments did not affect sperm motility (data not shown).

The osmolality of the culture media supplemented with or without urea and EGF was found to be 148.4, 147.7, 152.7, and 151.2 mOsm/l for control, EGF, 40 mg/dl urea, and EGF + urea groups, respectively. Using the hypo-osmotic swelling (HOS) test, the percentage of sperm cells with coiled tails (indicating functional and intact plasma membrane) was found to be 41.0%, 42.3%, 35.3%, and 38.3% for control, 10 ng/ml EGF, 40 mg/dl urea, and EGF + urea groups, respectively (Fig. 2f). The percentage of coiled-tail sperm obtained from the HOS test was increased by a 30-min incubation of 40 mg/dl of urea compared to the control and EGF group (*p* < 0.01). Representative images of TUNEL-positive sperm, non-capacitated sperm cells (F pattern), capacitated sperm cells (B pattern) and acrosome-reacted sperm cells (AR pattern) are shown in Fig. 2g–j. As seen in Fig. 2a–f, the EGF group (10 ng/ml) reversed the negative effect of urea on progressive sperm motility (*p* < 0.05), acrosome reaction (*p* < 0.05), capacitation (*p* < 0.001), sDF (*p* < 0.001) and HOS test score (*p* < 0.05).

**Figure 3:**
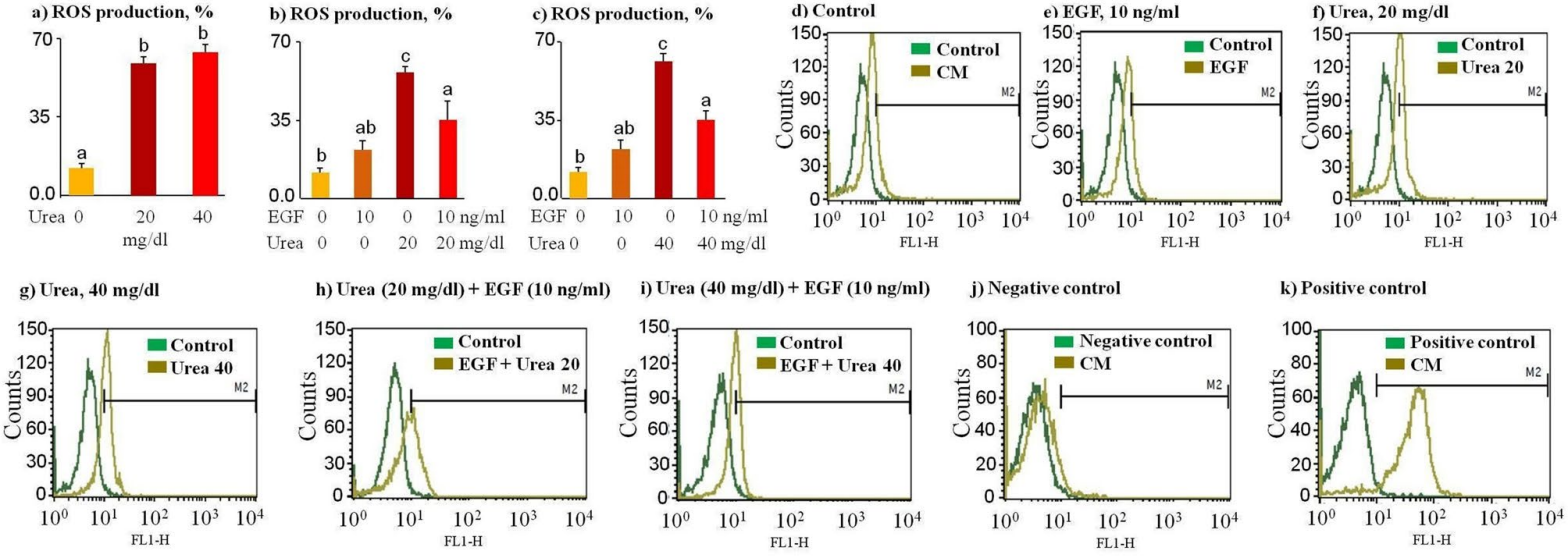
(**a**–**c**) ROS production by sperm in the presence of EGF and urea. Bovine sperm cells were pre-incubated for 30 min with various levels of urea (0, 20, and 40 mg/dl) in the presence or absence of EGF (10 ng/ml). (**d**–**i**) Good staining has been obtained, which has shown a marked increase in DCF fluorescence (FL-1) in sperm cells treated compared to control sperm cells, suggesting an increase in ROS production. (**j**–**k**) An aliquot of the unstained culture medium (TALP) was treated for 15 min with 10% H_2_O_2_ (positive control) or 1 mM N-acetyl-l-cysteine (ROS Inhibitor) as a negative control of the technique. The data was analyzed using the Tukey test (n = 3). *CM* culture medium, *Control* sperm incubation without EGF or urea, *EGF* sperm incubation with 10 ng/ml EGF, *Urea 20* sperm incubation with 20 mg/dl urea, *Urea 40* sperm incubation with 40 mg/dl urea, *EGF + Urea 20* sperm incubation with 10 ng/ml EGF and 20 mg/dl urea, *EGF + Urea 40* sperm incubation with 10 ng/ml EGF and 40 mg/dl urea. Numerical values are presented as mean ± S.E.M. Different letters (a, b, c) indicate significant differences between treatments at *P* < 0.05.

The data showed that a high level of urea increased ROS production (*p* < 0.01, Fig. 3a) compared to the control and EGF group. In addition, 10 ng/ml of EGF reduced (*p* < 0.01) urea-increased ROS production of sperm cells (Fig. 3b,c); however, sDF (*p* < 0.05, Fig. 2e) and ROS levels (Fig. 3b,c) in EGF-urea-pretreated sperm cells were still higher than in the control group.

### Pre-treatment of sperm with EGF and urea decreases the rate of fertilization and the subsequent development of embryos

**Figure 4:**
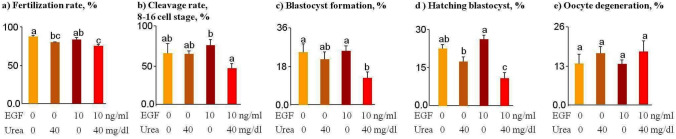
The result of in vitro fertilization in response to sperm pre-incubated with EGF or urea. Bovine sperm cells were pre-incubated with urea (40 mg/dl) for 30 min in the presence or absence of EGF (10 ng/ml). Data was obtained with a total of six replicates (n = 6, 28–36 embryos per replication) and analyzed using the Tukey test. Numerical values are presented as mean ± S.E.M. Different letters (a, b, c) indicate significant differences between treatments at *P* < 0.05.

Sperm pre-treated with urea (*p* = 0.04) reduced the rate of fertilized oocytes (2-PN) compared to the control group (Fig. 4a). A further decrease in fertilization rate was observed with insemination of oocytes with urea-EGF-pretreated sperm cells compared to insemination with urea-pretreated sperm (*p* = 0.007, Fig. 4a).

EGF- or urea-pretreated sperm cells did not change the cleavage percentage (8–16-cell stage, Fig. 4b). EGF-urea-pretreated sperm cells decreased (*p* < 0.05) the percentage of blastocyst formation (day 7) and hatching (day 9) compared to control, EGF, or urea groups (Fig. 4c,d). In the case of insemination with EGF-urea-pretreated sperm cells, the percentage of inseminated COCs entering the cleavage stage, the blastocyst stage and the hatching stage was decreased compared to the control group, the EGF group, or the urea group (*p* < 0.01, Fig. 4b–d).

### Sperm pre-treatment with a mixture of EGF and urea results in the expression of the apoptotic marker in the resulting blastocysts

**Figure 5:**
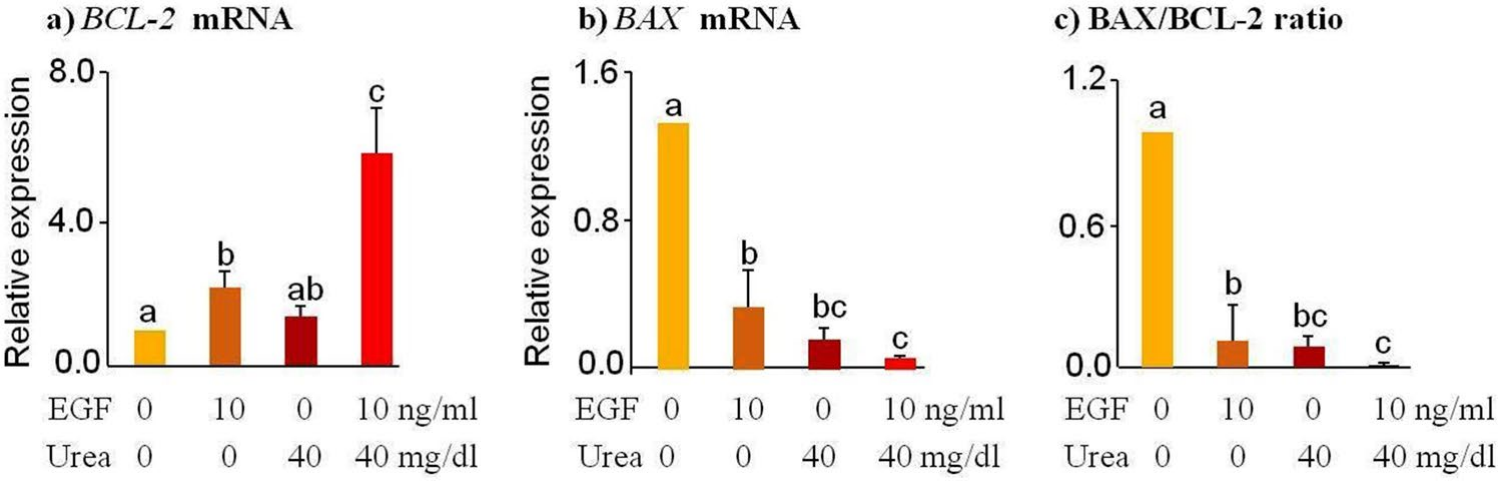
The gene expression in blastocysts derived from sperm pre-incubated with EGF or urea. Bovine sperm cells were pre-incubated with urea (40 mg/dl) for 30 min in the presence or absence of EGF (10 ng/ml). (**a**–**c**) Relative mRNA expression of *BCL-2* (anti-apoptotic gene) and *BAX* (pro-apoptotic gene) in day-9 blastocysts. Data was obtained with a total of six replicates (n = 6, 28–36 embryos per replication) and analyzed using the Tukey test. Numerical values are presented as mean ± S.E.M. Different letters (a, b, c) indicate significant differences between treatments at *P* < 0.05.

The mRNA expression of *BCL-2,* an anti-apoptotic gene, was up-regulated in embryos derived from EGF-incubated sperm cells (*p* < 0.01; Fig. 5a). The expression of *BCL-2* was increased in the blastocysts derived from EGF-urea-pretreated sperm cells compared to control, EGF, and urea groups (*p* < 0.001, Fig. 5a). The mRNA expression of the pro-apoptotic gene, *BAX,* was down-regulated in embryos derived from EGF- or urea-incubated sperm cells (*p* < 0.001; Fig. 5b). Moreover, *BAX* expression was down-regulated in embryos produced from EGF-urea-pretreated sperm cells compared to control (*p* < 0.001) and EGF groups (*p* = 0.04, Fig. 5b). In addition, the ratio of *BAX* to *BCL-2* was significantly decreased in embryos derived from EGF, urea, or EGF-urea-pretreated sperm cells (*p* < 0.001) compared to the control group (Fig. 5c).

### Relationship between ROS production, sperm parameters and oocyte competence

**Figure 6:**
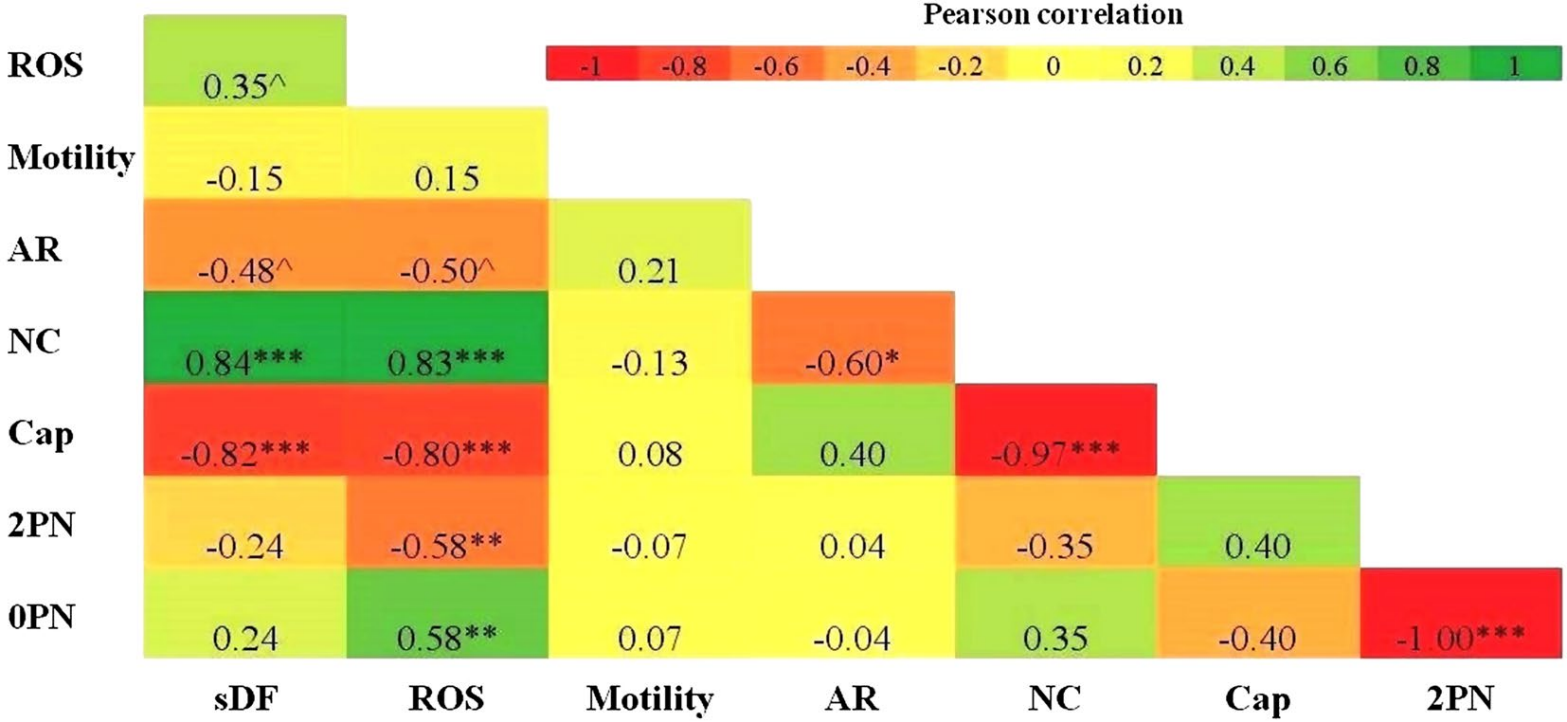
(**A**) Heatmap of pairwise correlations between variables. The Anderson–Darling test showed that data were normally distributed. Therefore, the Pearson correlation coefficient (as parametric correlation estimator) was used to assess the association between the two variables. The red and green boxes display negative and positive correlations, respectively. *ROS* reactive oxygen species, *sDF* sperm DNA fragmentation, *NC* non-capacitation, *AR* acrosome reaction, *PN* pronuclear, *Cap* capacitation, *Motility* progressive sperm motility. ^, *, **, and *** indicate *P* < 0.15, *P* < 0.05, *P* < 0.01, and *P* < 0.001, respectively. The scale bar shows the relationship between the variables.

As shown in Fig. 6, the Pearson correlation analysis showed that there was a correlation between sDF (the percentage of TUNEL-positive sperm cells) and capacitation (R = − 0.82, *p* < 0.001), non-capacitation (R = 0.84, *p* < 0.001), ROS production (R = 0.35, *p* < 0.15), and acrosome reaction (R = − 0.48; *p* = 0.10). ROS production was correlated with capacitation (R = − 0.80, *p* < 0.001), non-capacitation (R = 0.83, *p* < 0.001), 2-PN (R = − 0.58, *p* < 0.01), and acrosome reaction (R = − 0.50; *p* = 0.09) as shown in Fig. 6.

**Figure 7:**
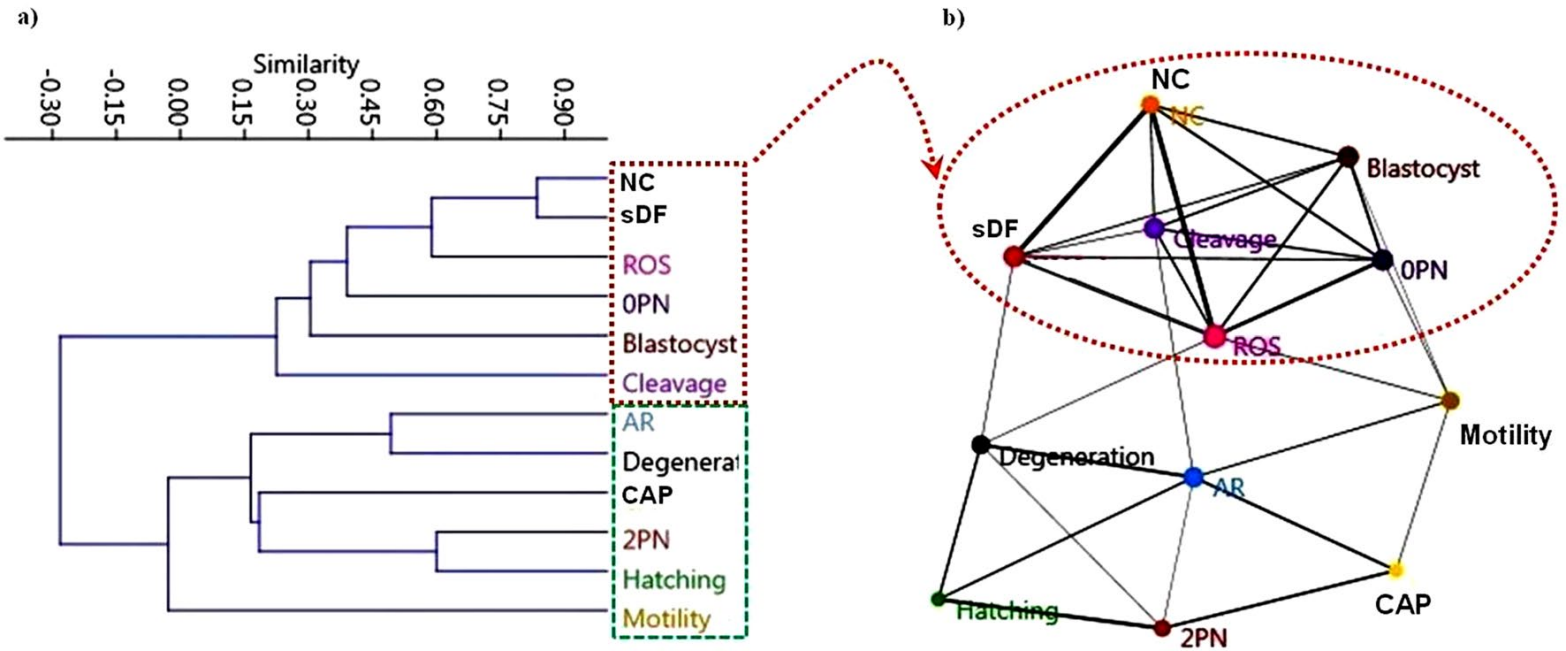
Study of hierarchical clustering (HCA) and network analysis. (**a**) The HCA analysis using the Pearson distance metric and un-weighted pair group method (UPGMA algorithm) was performed using PAST software to generate clustering patterns of sperm parameters, ROS production, and in vitro fertilization outcomes. Dashed color boxes (red and green) on the right side of the HCA show two distinct clusters. (**b**) Network analysis of the parameters of sperm and oocyte competence. All parameters are represented by circles of the network. Network analysis with visualization was performed by the PAST tool using the Fruchterman–Reingold algorithm as a force-directed layout algorithm. The Pearson correlation was chosen to determine the association between the edges and the nodes. The 50% threshold was the highest cut-off point in the visualization of the network in which there was no subsequent interaction between the variables. Nodes are parameters of sperm quality and oocyte competence. Edges represent the relationship between all variables. The size of the nodes and edges refers to the coefficient of clustering and the coefficient of correlation, respectively. Small nodes and thin edges indicate small values. Cluster and network analyses exhibited closeness between reactive oxygen species (ROS) production, cleavage rate, 0-PN rate, DNA fragmentation and blastocyst formation; red dashed box in part (**a**) and red dashed line in part (**b**). *sDF* sperm DNA fragmentation, *NC* non-capacitation, *AR* acrosome reaction, *PN* pronuclear, *CAP* capacitation, *Motility* progressive sperm motility.

The hierarchical clustering analysis (HCA) analysis identified two distinct clusters (Fig. 7a) such that the first cluster consisted of non-capacitation, sDF (TUNEL-positive sperm cells percentage), ROS production, 0-PN (oocytes that did not reach the 2-PN stage), blastocyst and cleavage rates (dashed red box in Fig. 7a). The second cluster consisted of acrosome reaction, oocyte degeneration, capacitation, 2-PN, hatching and progressive sperm motility rates (dashed green box in Fig. 7a).

In an attempt to visualize the relationship between factors, using a network analysis, we found closeness between non-capacitation, sDF, ROS production, 0-PN percentage, blastocyst formation, and cleavage rate (dashed red circle in Fig. 7b). The network analysis supported the findings of the HCA analysis, providing a deeper understanding of the relationship between ROS production, sperm parameters, and oocyte developmental competence. Moreover, the network analysis displayed that ROS had similarities to non-capacitation and 0-PN (darker lines in Fig. 7b). It was also noted that sDF had a strong connection to the non-capacitation rate.

**Figure 8:**
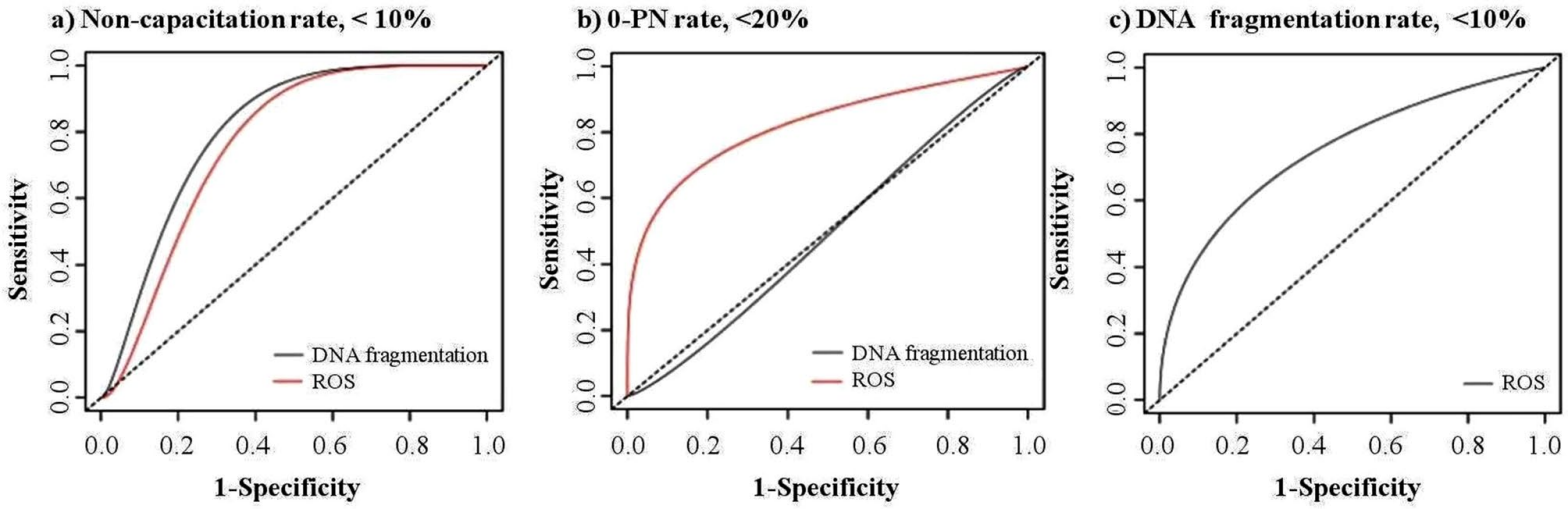
ROC analysis to describe the predictive power of the parameters to discriminate against low sperm quality. Using the easyROC web-tool, an analysis of the receiver operating characteristic (ROC) curve was done on the basis of the network analysis outputs that determined links between the sperm parameters. The analysis was carried out by determining the predictive power and the cut-off point of the network-detected parameters for the detection of high sperm quality by the area under the ROC curve (AUC). The optimal cut-off was determined by maximizing the Youden index. In order to address possible biases in the findings generated by assuming that any relationship between ROS production and non-capacitation and 0-PN rates could be linear, the results were divided into separate categories as follows: (1) low rate of non-capacitation (< 10%); (2) low rate of 0-pronuclear (0-PN, oocyte which did not reach the 2-PN stage, < 20%); and (3) low rate of DNA fragmentation (< 10%).

Finally, the ROC curve analysis defined ROS levels < 42% and < 31% as the optimal cut-off points for distinguishing sperm cells with no high non-capacitation rate (AUC 0.77, *p* = 0.04) and no high 0-PN rate (AUC 0.82, *p* = 0.006), respectively (Fig. 8 and Table 1). In addition, the AUC of the ROC analysis determined that ROS levels < 24.1% were the optimum cut-off point for the detection of low sDF (AUC 0.75, *p* = 0.04). The AUC analysis showed that sDF was good for the detection of sperm cells with low rates of non-capacitation (AUC 0.81, cut-off point of < 10.9%, *p* < 0.01).

**Table 1:** Discriminating power (the AUC analysis) of ROS for sperm non-capacitation and fertilization rates.

	AUC	*P* value	Optimal cut off point, %	Sensitivity	Specificity	Positive predictive value	Negative predictive value	Positive likelihood ratio (LR)	Negative likelihood ratio (LR)	AUC rank
**Non-capacitation, < 10%**										
sDF	0.81	0.01	< 10.9	100	60.0	77.8	100	2.5	0.0	1
ROS	0.77	0.04	< 42.0	100	60.0	77.8	100	2.5	0.0	2
**0-PN rate, < 20%**										
ROS	0.82	0.006	< 31.0	70.0	100	100	55.6	Inf	0.3	1
sDF	0.43	0.57	< 4.7	78.6	50.0	78.6	50.0	1.6	0.0	2
**sDF, < 10%**										
ROS	0.75	0.04	< 24.1	50.0	100	100	50.0	Inf	0.5	1

In order to identify the best predictive variables for predicting a high rate of fertilization, a receiver operating characteristic (ROC) curve analysis was performed using the easyROC web-tool. Optimal cut-off points were determined by maximizing the Youden index. The results were divided into distinct groups as follows: (1) low rate of non-capacitation (< 10%) and (2) low rate of 0-PN, oocyte which did not reach the 2-PN stage (< 20%). In addition, the optimal level of ROS for detecting the incidence of low DNA fragmentation (< 10%) was estimated using the AUC analysis. *sDF* sperm DNA fragmentation, *NC* non-capacitation, *AR* acrosome reaction, *PN* pronuclear.

## Discussion

In this study, high-level urea (40 mg/dl) increased ROS production and sDF but decreased progressive sperm motility, capacitation and acrosome reaction. Urea has been shown to have functional amino groups and to serve as an active nucleic acid denaturant^22,23^. As a consequence, urea can have the potential to damage sperm DNA. It has been reported that compared to sperm cells derived from fertile bulls, those obtained from infertile bulls had a 1.6-fold increase in sDF^24^. Beran et al.^14^ found that high urea concentrations (> 26 mg/dl) in cow’s cervical mucus significantly reduced sperm viability, suggesting that there could be a correlation between damaged sperm DNA and reduced embryo production^25^. sDF is characterized as a single or double strand breaks in nuclear DNA and is associated with heterospermic output and sperm quality, resulting in a possible loss/alteration of genetic material and adverse pregnancy outcomes^26^. Indeed, oocytes do not recognize DNA-damaged sperm^26^. In addition, embryos produced from high sDF sperm may be lost at a later stage when proteins are likely to be required; these proteins are missing due to the DNA/gene breakup needed to supply the essential proteins^26^. The threshold at which sDF has a negative effect on fertility differs between species, e.g. pigs: 6%, bulls 10–20%, horses: 28%, humans: 25–30%^26^. It has been reported that sperm cells with a TUNEL index lower than 20% do not influence the artificial insemination outcome^27^. However, the chance of success of artificial insemination was 10% higher than the average for bulls with a sperm TUNEL index of less than 4.8%^27^. In this study, TUNEL-positive sperm cells in the urea and urea + EGF groups were 4.5% which was not beyond the threshold reported by others^27^. This implied that the reduced developmental competence of embryos derived from urea-EGF-pretreated sperm cells was not due solely to a slight increase in sDF levels.

In addition, the urea-incubated sperm cells had a low HOS test score compared to the control and EGF groups, but the EGF reduced the adverse effect of urea on the HOS challenge. In other words, urea canceled the positive effect of the EGF on HOS-positive sperm cells. The HOS test evaluates the ability of the sperm plasma membrane to transport water when confronted with hypo-osmotic solutions. This thereby induces sperm swelling and expansion of the plasma membrane^28^. If there is no transport of water, it can be inferred that the sperm plasma membrane is not biochemically active and cannot function during the fertilization process^28,29^. Since HOS coiling is closely associated with motility, normal morphology and livability^29^, fertilization does not occur when the sperm plasma membrane is physically intact but biochemically inert. In men, infertile sperm cells have been reported to have a low HOS test score and a low pregnancy rate^30^. Current data implied that urea could reduce sperm motility, alter sperm plasma membrane, and reduce the HOS test score, resulting in a reduced fertility.

As described above, more ROS was produced by sperm pre-treated with 40 mg/dl of urea. Urea has been shown to induce mitochondrial ROS production in primary human aortic endothelial cells via increased NADPH oxidase activity, which generates ROS through the transfer of electrons from NADPH to molecular oxygen^31^. D'Apolito et al. stated that the mitochondrial electron transport chain is a source of urea-induced ROS production^31^. At physiological level, ROS has been reported to enhance sperm capacitation and acrosome reaction^8,9^, but high levels of ROS can lead to lipid peroxidation of sperm and, ultimately, sperm integrity degradation, low sperm motility and damage to sperm DNA^32,33^. Unsaturated lipid moiety and protein channels in the middle part of the sperm have been shown to be extremely vulnerable to ROS-induced damage^34^. Shamsi et al*.*^35^ reported that increased ROS production could damage mitochondrial DNA, ATP production, and energy availability, leading to a reduction in sperm motility and fertilizability. Zorn et al.^36^ stated that increased seminal ROS is associated with decreased sperm fertilizability and lower rates of pregnancy following IVF. It has been shown that ROS can impair the necessary fertilization processes, such as capacitation^37^, acrosome reaction and sperm-oocyte fusion^32,38^. In fact, oxidative stress during capacitation has been shown to reduce the surface expression of the zona pellucida-receptor arylsulphatase A in human sperm. As a result, the sperm binding to the zona pellucida and its fertilizability could decrease^32^. In terms of decreased fertility of dairy cows due to increased BUN, the present results implies that exposure of post-thaw sperm to a high level of urea increases a high level of ROS, which can lead to a reduction in sperm fertility.

In this study, pre-treatment with EGF (10 ng/ml) did not affect sperm capacitation and acrosome reaction. In line with our findings, Naz and Kaplan^39^ reported that incubation with EGF (0.1–10 ng/ml) did not affect human sperm capacitation and acrosome reaction. Lax et al*.*^7^ reported that incubation with EGF (10 or 100 ng/ml) reduced acrosomal exocytosis. However, it was found that low doses of EGF (1 or 0.1 ng/ml) induced acrosome reaction in bovine sperm^7^.

In this study, we found that the EGF molecule completely reversed the negative effects of urea on progressive sperm motility, capacitation, and acrosome reaction. In a previous study, Humphrey et al.^40^ reported that the EGF abolished hypoxia-induced apoptosis in human trophoblasts by selective phosphorylation of the pro-apoptotic protein Bcl2-associated death promoter. Sancho et al.^41^ reported that EGF inhibited the production of ROS to protect cells from apoptosis. ROS has been reported to enhance sperm function by inducing redox-regulated cAMP-mediated pathways that are essential for sperm capacitation^42,43^. The results showed that the EGF was capable of alleviating the negative effects of urea on sperm quality by reducing ROS production and sDF, reflected in restored sperm motility, capacitation and acrosome reaction.

With regard to the protective impact of EGF against urea-reduced sperm quality, we assumed the role of EGF to reduce the potential negative effects of urea on the developmental competence of oocytes inseminated with urea-pretreated sperm. However, ROS production and sDF levels in EGF-urea-pretreated sperm cells were still higher than control and were concomitant with reduced fertilization, cleavage, blastocyst and hatching rates in these sperm cells. In fact, overproduction of ROS can overcome sperm antioxidant defense systems, resulting in increased sDF and decreased sperm function due to peroxidative damage to the sperm membrane, leading to lower fertility^44,45^. Similarly, Humphrey et al.^40^ reported that the EGF reduced the level of hypoxia-induced apoptosis in trophoblast cells while the level of apoptosis was still higher than the control point. They suggested that EGF protection against apoptosis is incomplete, a finding that is consistent with our results. This can, at least in part, explain the increased production of ROS and sDF in EGF-urea-pretreated sperm cells, resulting in reduced fertilization rate, cleavage rate, blastocyst formation, and hatching rate compared to the control group. ROS has been reported to cause sperm DNA fragmentation^31^, which results in compromised pronuclear and blastocyst formation and decreased pregnancy following IVF^36,46^. Watanabe et al.^47^ injected murine oocytes with dithiothreitol-pretreated sperm and found that the resulting embryos did not develop into a live birth stage, implying a carryover effect of sperm quality on the resulting embryo. There is a growing body of evidence that paternal factors and oxidative damage to sperm can contribute to early abnormal cleavage^48,49^, embryo nuclear fragmentation and mitotic arrest, and may alter the transcript abundance of genes in embryos^50,51^. It has also been reported that embryos derived from sperm cells exposed to high level of ROS did not grow beyond 4-cell stage^48^. These findings indicate the potential role of sperm quality in embryonic failure or death ^52^. Importantly, it should be remembered that more research is needed as the reduced developmental competence may arise from other urea-activated signaling pathways which have been shown to interfere with EGFR signaling^19^.

Results showed that the *BCL-2* expression (an anti-apoptotic marker) was up-regulated and *BAX* expression (a pro-apoptotic marker) was down-regulated in day-9 blastocysts derived from EGF-pretreated sperm compared to control and urea groups. Moreover, when sperm cells were pre-treated with both EGF and urea, *BCL-2* expression was significantly increased and *BAX* expression was completely inhibited. This was reflected in the reduced *BAX* to *BCL-2* ratio, which can be used as an indicator for determining the viability or apoptosis of bovine embryos^53^. In fact, the expression of apoptotic genes (*BAX* and *BCL-2)* is regulated by EGFR signaling, which plays a critical role in the regulation of apoptosis^54^. Zhao et al.^19^ reported that both urea and EGF use EGFR transactivation and induce the expression of the immediate-early genes. As the head and mid-piece of bovine sperm express EGFR^6,7^, we deduced that urea would interact with the EGF/EGFR system present in sperm and that this effect could be passed on to subsequent blastocysts. ROS has been reported to induce up-regulation of genes that code anti-oxidant proteins in terms of increased oxidative stress^8^. Of interest, BCL-2 has been reported to have anti-oxidant effects^55^. Current data indicate that the insemination of oocytes with urea-EGF-pretreated sperm cells resulted in an up-regulation of *BCL-2* and a down-regulation of *BAX* in the resulting blastocysts, such adaptation of blastocysts to carry-over effects of urea-EGF-damaged sperm needs further investigation^56^.

In an attempt to describe the relationship between sperm parameters, a network analysis identified the proximity of ROS production or the level of DNA fragmentation to non-capacitation and 0-PN (thicker lines, edges, in Fig. 7b). Moreover, based on the Pearson correlation analysis, we found a strong and positive association between ROS production and non-capacitation and 0-PN rates (Fig. 6). Accordingly, this strong relationship was pronounced in the higher non-capacitation and 0-PN rates of the EGF-urea-pretreated sperm group. Zorn et al.^36^ stated that elevated seminal ROS levels are associated with decreased sperm fertilizability, damage to sperm membrane, and adversely affect the necessary fertilization processes, such as capacitation^37^ and sperm-oocyte fusion^32,38^.

As described above, the network analysis identified a close link between ROS production, non-capacitation rate, and 0-PN rate. Similarly, the ROC analysis also showed that ROS production was the best predictor for the detection of sperm cells with a high capacitation rate (AUC 0.77) and a high 2PN rate (AUC 0.82). The AUC analysis also determined that ROS levels of < 42% and < 31% were the optimum cut-off points for the detection of sperm cells without high non-capacitation and 0-PN rates, respectively. Using the ROC analysis, Homa et al.^57^ reported that values below 24.1 (RLU/s/10^6^ sperm) are the optimal cut-off for ROS production, which is likely to imply the basal physiological level of ROS in human semen^36^. The present finding showed that the ROS production threshold for the incidence of high 0-PN rates was lower than that for the incidence of high non-capacitation rates. It was noted that the elevated ROS production of urea-EGF-pretreated sperm cells surpassed the cut-off threshold obtained from the AUC analysis, which corresponded to a low rate of capacitation and fertilization in these sperm cells.

Taken together, the present study suggests that the protective effects of EGF against urea-reduced sperm quality are incomplete and that the unsolved interaction between urea and EGF on subsequent fertilization and developmental competence of oocytes inseminated with these sperm cells should be further investigated.

## Methodology

All the reagents were obtained from Sigma-Aldrich Inc., St. Louis, MO, USA, unless otherwise stated.

### Statement of ethics and availability of data

Animal experiments were carried out according to the Guiding Principles for the Care and Use of Research Animals Promulgated by the Isfahan University of Technology, Iran. The protocols and methods were approved by the Committee on the Ethics of Animal Experiments of the Isfahan University of Technology (No. 390132). The datasets of the present study are available from the corresponding authors on reasonable request. The study was carried out in compliance with the ARRIVE guidelines.

### Preparation of bovine sperm and determination of effective doses of EGF and urea

Frozen semen straws (n = 5) from Holstein bulls (n = 4) were thawed in a water bath at 35 to 37 °C for 30 s, pooled, and washed in 15 Falcon tubes using a density gradient solution^58^ (80/40, PureCeption, SAGE, USA). The centrifugation was carried out at 800×*g* for 15 min, and then the pellet was diluted with 3 ml of tyrode albumin lactate pyruvate (TALP) solution and centrifuged for the second time at 300×*g* for 3 min. The resulting pellet was immersed in the TALP solution (3 ml) and the sperm concentration was measured using a hemocytometer, adjusted to 0.5 million per ml, and incubated with different concentrations of EGF and urea. The motility, progressive motility and HOS test score for post-thaw sperm cells were 55 ± 2.5, 40 ± 2.1%, and 41.1 ± 0.5, respectively.

The different doses of EGF (0, 10, 100, and 1000 ng/ml) and urea (0, 20, and 40 mg/dl) were used to identify the effective doses of EGF and urea (this experiment was replicated five times independently, n = 5). It was observed that 10 ng/ml of EGF significantly induced progressive sperm motility and 40 mg/dl of urea increased DNA fragmentation and decreased progressive sperm motility (Fig. 1).

For next experiments, sperm cells were pre-treated with urea (40 mg/dl), EGF (10 ng/ml) or a mixture of urea (40 mg/dl) and EGF (10 ng/ml) for 30 min at 38.5 °C under 5.5% CO_2_ (n = 6, six replicates per treatment). After this step, sperm cells were washed using Tyrode’s medium containing lactate, pyruvate, and HEPES (TL-HEPES) and subsequently used for in vitro fertilization (IVF). At this point, progressive sperm motility was subjectively assessed by a computer-assisted semen analyzer (CASA) visual estimation as described by others^59^. In brief, samples of semen were deposited in a 20-µm-deep chamber (SC20-01-04-B, Leja, GN Nieuw-Vennep, Netherlands) which was pre-heated to 37 °C on a hot plate. Sperm motility was evaluated using a CASA system (SMAS, DITECT, Tokyo, Japan) based on digital images obtained using × 10 negative-phase contrast microscope (E200, Nikon, Tokyo, Japan). The sperm count measured was at least 200 per sample.

Different urea concentrations (0, 20, and 40 mg/dl equivalent to 9.3 and 18.7 mg/dl of BUN, respectively) were chosen to simulate BUN physiological values of healthy dairy cows fed low or high dietary protein^12,13,60^. Importantly, there is a high association between urea concentrations in the blood and FRT fluids in dairy cows^11,13^. In the present study, the preliminary experiment showed that sperm pre-treated with a high level of urea had significantly higher DNA fragmentation rates (Fig. 1c). For further experiments, the highest level of urea (40 mg/dl equivalent to 18.7 mg/dl of BUN) known to be associated with reduced fertility in healthy dairy cows fed high dietary protein was selected. Increased urea concentrations above 19 mg/dl in both blood and FRT fluids are associated with reduced pregnancy rates in healthy dairy cows^10,11,61^.

### Evaluation of bovine sperm capacitation and acrosome reaction

Using a sperm-washing medium supplemented with albumin, sperm cells were capacitated in the absence (control) or presence of urea (40 mg/dl), EGF (10 ng/ml), or the presence of both urea (40 mg/dl) and EGF (10 ng/ml) for 30 min at 37 °C and 5% CO_2_ (n = 5, five replicates per treatment). Chlortetracycline (CTC) staining was used to determine the percentage of non-capacitated, capacitated and acrosome-reacted sperm cells as described in other studies^62,63^**.**

### Hypo-osmotic swelling (HOS) test of bovine sperm

The HOS test was conducted as defined by others^28^. To this aim, 0.1 ml of the semen sample was mixed with 1.0 ml of a hypo-osmotic solution containing EGF, urea, or a mixture of EGF and urea (n = 4). The hypo-osmotic swelling solution (100 mOsm/kg) was made by dissolving 0.9 g of fructose and 0.49 g of sodium citrate in 100 ml of distilled water. After 30-min incubation at 37 °C, at least 100 sperm cells were analyzed by phase-contrast microscopy to measure sperm tail changes (swollen sperm), which were reported as a percentage of all sperm observed. The sperm cells with swollen tail were counted as HOS-positive sperm.

### Flow‐cytometric technique of reactive oxygen species detection

Dichloro-dihydro-fluorescein diacetate (DCFH-DA, a cell-permeable ester)^64,65^ was used to evaluate ROS production with 3 replicates (n = 3). DCFH-DA (10 µM) was added to the sperm suspensions (2 × 10^7^ spermatozoa/ml) and incubated at 37 °C for 30 min in the dark. Next, flow-cytometric quantification was conducted in the BD FACSCalibur flow-cytometer (Becton Dickinson, San Jose, CA) using BD CellQuestPro software (BD Biosciences, San Jose, CA, USA). The excitation and emission wavelengths were set at 490 nm and 540 nm, respectively. Results were expressed as relative fluorescence unit (RFU) for 1.5 × 10^4^ sperm cells. An aliquot of the unstained culture medium (TALP) was treated for 15 min with 10% H_2_O_2_ (positive control) or 1 mM N-acetyl-l-cysteine (ROS Inhibitor) as a negative control of the technique.

### TUNEL assay

TUNEL assay was performed using a commercial detection kit (Apoptosis Detection System Fluorescein; Promega, Mannheim, Germany) to determine sperm DNA fragmentation as instructed by the manufacturer. At least 400 sperm cells were randomly selected and immediately analyzed using an Olympus fluorescent microscope (BX51, Tokyo, Japan) with proper emission filters (460–470 nm) at 100 × magnification. Sperm cells with a green fluorescent color were considered TUNEL-positive cells. Positive controls (incubation with DNase A) and negative controls (without TdT enzyme) were developed for each batch of slides to standardize the assay^66–68^. The TUNEL assay was performed five times (n = 5) and at least 400 sperm cells were counted at each of the 10 fields of vision. The proportion of positive TUNEL cells in each treatment was averaged over 5 replications.

### Cumulus-oocyte complex collection and in vitro embryo production (IVP)

Bovine ovaries (approximately 600 COCs) were collected from a local slaughterhouse and transported to the laboratory at approximately 30 °C within 2 h at about 30 °C using a thermo box containing 0.9% NaCl, 0.1% penicillin, and streptomycin. Antral follicles (7–8 mm in diameter) were isolated from ovaries containing active corpus luteum and kept at 37 °C in HEPES-TCM-199 (tissue culture medium; Invitrogen, Carlsbad, CA, USA).

Cumulus-oocyte complexes (COCs) with at least five cell layers were selected using a stereomicroscope (Olympus, Tokyo, Japan) and transferred to HEPES-TCM-199 supplemented by 50 mg/ml of kanamycin and 50 mg/ml of heparin. Intact COCs were then washed twice in HEPES-TCM199 and randomly put in groups of 10 in 50-µl droplet of maturation medium containing bicarbonate-buffered TCM199 supplemented by 10% (w/v) of heat-inactivated fetal bovine serum (FBS, BioWhittaker, Walkersville, MD, U.S.A.) and 20 IU/ml follicle-stimulating hormone (FSH) in 35 mm cell culture dishes (Falcon brand, BD Biosciences, San Jose, CA, USA) for 24 h at 38.5 °C under 5.5% CO_2_, 20% O_2_, balanced N_2,_ and maximum humidity under mineral oil. The IVM was conducted for 24 h, according to previous studies^17,18^.

After IVM, approximately 600 COCs (from 60 cows) were assigned to different treatments: (1) the control group in which oocytes were inseminated by untreated sperm cells (n = 140 oocytes, through 10 replicates, each replication (drop) containing 10–15 oocytes); (2) the EGF group in which oocytes were inseminated by sperm cells pre-treated with 10 ng/ml EGF for 30 min (n = 150 oocytes, 10 replicates, each replicate (drop) containing 10–15 oocytes); (3) the urea group in which oocytes were inseminated by sperm cells pre-treated with 40 mg/dl urea (equivalent to 18.7 mg/dl BUN) for 30 min (n = 150 oocytes in 10 replicates, each replicate (drop) containing 10–15 oocytes); and (4) EGF-urea group in which oocytes were inseminated by sperm cells pre-treated with a mixture of 40 mg/dl of urea and 10 ng/ml of EGF for 30 min (n = 160 oocytes in 10 replicates, each replicate (drop) containing 10–15 oocytes).

Follicular urea levels were determined using an enzymatic method on the Hitachi 917 auto-analyzer (Roche Diagnostics, Mannheim, Germany). On average, urea levels were found to be 17.82 ± 3.46 mg/dl, ranging from 10 to 26 mg/dl. It is, therefore, important to explore the outcomes of IVF in which COCs are derived from follicles with different levels of urea, and sperm cells are pre-treated with EGF/urea.

For IVF and IVP, the oocytes were inseminated by sperm at a final concentration of 1 × 10^6^/ml in the IVF medium^17,18^. COCs insemination was performed in the IVF medium, supplemented by BSA in 35 mm cell culture dishes (Falcon brand, BD Biosciences, San Jose, CA, USA) at 38.5 °C under 5% CO_2_ in air. Developing embryos were scored using a stereomicroscope (Olympus, Tokyo, Japan) as 2-pronuclear (2-PN) zygotes (18 h after insemination); 8–16 cell stage (day 3); blastocyst stage (day 7); and hatching stage (day 9). Fertilization was verified by 2-PN formation, 18 h after insemination^69^. All developmental parameters, including the percentage of 2PN, cleavage rate, and blastocyst formation, were determined in relation to the initial number of COCs cultured in the maturation medium. In order to examine the formation of PN, some oocytes were fixed using 1.25% glutaraldehyde in phosphate-buffered saline solution (PBS) containing 25 µl of Triton X-I00 and stained with Hoechst 33342 in glycerol solution (0.0 l mg Hoechst in 1 ml of PBS + 9 ml of glycerol). Oocytes with cytoplasmic degeneration were classified as degenerated by day 3 after insemination^70^. Six pools of seven embryos (day 9, six replicates, n = 6) from each treatment were stored in the RNeasy lysis buffer (RLT; Qiagen) supplied with 2-mercaptoethanol (Sigma-Aldrich) at − 70 °C for qRT-PCR analysis.

### Extraction of RNA, reverse transcription, and qRT-PCR

RNA extraction and cDNA synthesis were conducted as previously reported^18,71^. In short, total RNA was isolated using the RNeasy Micro kit (Qiagen, Mississauga, Ontario, Canada), then incubated with 2 µl DNase I (Ambion, Streetsville, Ontario, Canada) at 37 °C for 15 min, and then heated to 75 °C for 5 min to inactivate DNase I. The WPA Biowave spectrophotometer (Cambridge, United Kingdom) was used to determine the quality and quantity of RNA at 260 and 280 nm absorbances. As a negative control, the cDNA synthesis was performed by omitting the Reverse Transcriptase for each sample. This negative control assesses the amount of DNA contamination present in the preparation of the RNA. The extracted total RNA was stored at − 80 °C in the RNA storage solution (Ambion, Austin, TX, USA) prior to use for the cDNA synthesis. The synthesized cDNA was stored at − 30 °C. The qRT-PCR was carried out in line with the previous studies^18,71^.

Reverse transcription (n = 6) was performed at 25 °C for 10 min, 42 °C for 1 h and 70 °C for 10 min. qRT-PCR was done using 1 µl of cDNA (50 ng), 5 µl of SYBR Green/0.2 µl ROX qPCR Master Mix (2X) (Fermentas, Germany) and 1 µl of forward and reverse primers (5 pM) adjusted to a total volume of 10 ml with nuclease-free water. The *ACTB* was used as a reference gene. Every biological replicate (i.e. the same cDNA sample) pipetted into three wells to eliminate technical errors. As a general control for extraneous nucleic acid contamination, a series of negative samples (without cDNA, but with primers) was taken in conjunction with the test samples. A limit (e.g. < 36 CT) for each corresponding gene was then set based on the CT observed for the negative samples (e.g. 36 CT). The rate of expression was normalized to that of the reference gene. The relative fold differences in the expression of the *BAX* and *BCL-2* genes were determined using the 2^−ΔΔCt^ method^72,73^. The bovine primers used in qRT-PCR are shown in Table 2.

**Table 2:** Bovine primers used in qRT-PCR.

Gene		Sequence of nucleotide (5′–3′)*	Tm (℃)	Product size, bp	Accession No
*BAX*	F	AGCGAGTGTTCTGAAGCG	61		AC_000175.1
	R	CCCAGTTGAAGTTGCCG		182	
*BCL-2*	F	CCTTCTTTGAGTTCGGAG	60		AC_000181.1
	R	CCTTCAGAGACAGCCAG		121	
*ACTB*	F	CCATCGGCAATGAGCGGT	60		AC_000182.1
	R	CGTGTTGGCGTAGAGGTC		146	

*Tm* melting temperature, *F* forward, *R* reverse.

### Statistical analysis

The normality of the data was confirmed by the Anderson–Darling test (EasyFit software, version 5.6, MathWave Technologies, Spokane, WA, USA). Statistical analysis was performed by StatView 5.0 software (SAS Institute Inc., Cary, NC). Data from DNA fragmentation (n = 5), acrosome reaction (n = 5), capacitation (n = 5), progressive sperm motility (n = 5) and ROS production and HOS test (n = 3) were analyzed using the one-way ANOVA analysis, followed by Tukey’s post hoc multiple comparison test. Also, the one-way ANOVA analysis, followed by Tukey’s post hoc multiple comparison test, was used to analyze data on gene expression (six replicates, n = 6). The average statistical power of this experiment was sufficient at 95.4 ± 5.7%, ranging from 89.4 to 100%. Data were reported as mean ± S.E.M. P values of less than 0.05 were considered statistically significant.

Bivariate analysis was conducted using the Pearson correlation to determine the relationship between sperm parameters, ROS production, and oocyte competence. The HCA analysis using Pearson distance metric and un-weighted pair group method algorithm (UPGMA) was conducted using PAST software (accessible at: http://folk.uio.no/ohammer/past) to generate clustering patterns of sperm parameters, ROS production, and oocyte competence parameters.

The network analysis was performed using the Pearson similarity index and the Fruchterman–Reingold algorithm as a force-directed layout algorithm. This algorithm organizes a network based on the strength of the link between nodes^74^. Pearson correlation and network mapping analysis were performed using PAST software. Based on the network outputs that defined a connection between sperm parameters, the ROC curve analysis was performed using the easyROC web-tool (accessible at: http://www.biosoft.hacettepe.edu.tr/easyROC/)^75^. This was done to identify the predictive power and the cut-off point of the network-detected parameters for the detection of high sperm quality by the area under the ROC curve (AUC). The optimal cut-off was determined by maximizing the Youden index. In order to cope with potential biases in the results produced by assuming that any relationship between ROS production and non-capacitation rate and 0-PN rate could be linear, the results were divided into separate groups as follows: (1) low rate of non-capacitation (< 10%); (2) low rate of 0-pronuclear (0-PN, oocyte which did not reach the 2-PN stage, < 20%); and (3) low rate of DNA fragmentation (< 10%).
